# Safety evaluation of the excessive intake of *Bacillus subtilis* C-3102 in healthy Japanese adults: A randomized, placebo-controlled, double-blind, parallel-group, comparison trial

**DOI:** 10.1016/j.toxrep.2019.11.009

**Published:** 2019-11-21

**Authors:** Misaki Hatanaka, Hiroki Kanzato, Ryoko Tsuda, Isao Nadaoka, Masaaki Yasue, Tomohiro Hoshino, Shin-ichiro Iio, Tsuyoshi Takara

**Affiliations:** a ASAHI CALPIS WELLNESS Co., Ltd., 2-4-1 Ebisuminami, Shibuya-ku, Tokyo 150-0022, Japan; b ORTHOMEDICO Inc., 2 F Sumitomo Fudosan Korakuen Bldg., 1-4-1, Koishikawa, Bunkyo-ku, Tokyo, 112-0002, Japan; c Medical Corporation Seishinkai, Takara Clinic, 9F Taisei Building, 2-3-2, Higashi-gotanda, Shinagawa-ku, Tokyo, 141-0022, Japan

**Keywords:** *Bacillus subtilis* C-3102, Gut microbiota, Bone mineral density, Excessive intake, Safety evaluation

## Abstract

**Objective:**

Continuous intake of *Bacillus subtilis* C-3102 (*B. subtilis* C-3102) has been reported to modulate the gut microbiota and increase the bone mineral density of the femur in healthy adults. This study aimed to evaluate the safety of excessive *B. subtilis* C-3102 intake through a randomized, placebo-controlled, double-blind, parallel-group study.

**Method:**

A total of 69 individuals provided an informed consent, and 44 subjects who met the inclusion criteria were allocated to either the *B. subtilis* C-3102 (C-3102 group, *n* = 22) or the placebo group (P group, *n* = 22). All subjects took 18 tablets containing either containing *B. subtilis* C-3102 or placebo per day for 4 weeks with water and without chewing. Subjects in the C-3102 group consumed 4.8 × 10^10^ colony forming units (cfu) per day. Physical examination, urinalysis, blood analysis, records of subjective symptoms, and a medical questionnaire administered by a clinical trial physician were performed to determine the safety of test tablets. Furthermore, bone mineral density was measured.

**Results:**

The final analysis included data from 22 subjects (9 men, 13 women; age, 46.1 ± 13.8 years) in the C-3102 group and 22 subjects (9 men, 13 women; age, 46.1 ± 13.5 years) in the P group. The results revealed no medical-related problems in both C-3102 and P groups.

**Conclusion:**

This study proved the safety of 4-week continuous consumption of an excessive amount of *B. subtilis* C-3102 tablets.

## Highlights

• Excessive intake of *B. subtilis* C-3102 leads to unchanged in the medical condition.
• Excessive intake of *B. subtilis* C-3102 does not worsen bone mineral density.
• Excessive intake of *B. subtilis* C-3102 was safe under the study condition.

## 1 Introduction

Approximately 100 trillion bacteria and microbes reside in the intestines of humans, forming diverse colonies [1]; these are referred to as gut microbiota. The composition and metabolism of gut microbiota are influenced by dietary habitation [2], stress [3], aging [4], and other factors, and are also affected by several diseases [5], which lowers the quality of life. Therefore, improving the intestinal environment is essential for health maintenance and promotion.

A previous clinical study demonstrated that an intake of 9.0 × 10^8^ cfu of *Bacillus subtilis* C-3102 (hereinafter referred to as C-3102^1^) for 8 days significantly reduced para-cresol concentration and coliform bacterial counts in feces and significantly increased the relative abundance of genera *Bifidobacterium* [6]. Moreover, the intake of 2.2 × 10^9^ cfu of C-3102 per day for 8 weeks in healthy subjects having loose stools significantly lowered the Bristol scale score and stool frequency and modulated the gut microbiota [7]. Thus, the consumption of C-3102 is considered to enhance human health by improving the intestinal environment.

Recently, postmenopausal women who took 3.4 × 10^9^ cfu of C-3102 per day for 24 weeks had improved bone mineral density (BMD^2^) in the femur [8]. In addition, the relative enrichment of *Bifidobacterium* significantly increased at 12 weeks of treatment compared with that at the baseline in the C-3102 group [8]. Furthermore, the relative abundance of *Fusobacterium* was significantly lower in the C-3102 group at 12 and 24 weeks of treatment compared with that at the baseline [8]. These data suggested that C-3102 modulated the gut microbiota and improved BMD by inhibiting bone resorption in healthy postmenopausal women. In the same study, safety problems associated with the intake of 3.4 × 10^9^ cfu of C-3102 per day were not observed for 24 weeks; however, studies investigating the safety of excessive intakes of C-3102 were limited [8]. Hence, this study was conducted to investigate the safety of consuming 4.8 × 10^10^ cfu of C-3102 per day and its influence on BMD.

## 2 Materials and methods

### 2.1 Study design

A randomized, double-blind, placebo-controlled, parallel-group study was conducted at Takara Clinic (Medical Corporation Seishinkai, Tokyo Japan). The study protocol was approved by the ethical committee at Takara Clinic (Tokyo, Japan) on February 5, 2018 (approval ID: 1802-1712-AK01-01-TC). We conducted this study in accordance with the principles of the Declaration of Helsinki (2013) and the ethical guidelines for medical and health research involving human subjects of Japan and broader medical ethics. The study protocol was registered at the University Hospital Medical Information Network Clinical Trials Registry (UMIN000031218).

### 2.2 Subjects

This study included healthy Japanese subjects. The exclusion criteria were as follows: (1) having any medical history of malignant tumor, heart failure, or myocardial infarction; (2) undergoing treatment for arrhythmia, liver disease, kidney disease, cerebrovascular disease, rheumatism, diabetes mellitus, hyperlipidemia, hypertension, irritable bowel syndrome, osteoporosis, or any other chronic diseases; (3) consuming “Food for Specified Health Uses” and/or “Foods with Function Claims” daily; (4) regularly using medications such as herbal medicines and/or supplements, particularly anticoagulants, such as warfarin; (5) having histories of allergic reactions to medications and/or products associated with the study substances, particularly soybeans and fermented soybeans; (6) being lactose intolerant; (7) being pregnant, lactating, or expecting/planning to be pregnant during the study period; (8) participating in another clinical study within the last 3 months prior to signing the study’s informed consent form; and (9) identified as ineligible to participate in this study by the primary physician.

All subjects were recruited through the website (https://www.go106.jp/) managed by ORTHOMEDICO Inc. (Tokyo, Japan) between February and April 2018 and enrolled in this study. The study protocol was comprehensively explained to the subjects, who provided written informed consent prior to participation in the study at ORTHOMEDICO Inc. office. Women with increased BMD aged 50–69 years were recruited to evaluate the safety of C-3102 [8]. No subject was part of the sponsors or funding companies. The intervention period was from April to May 2018.

### 2.3 Sample size determination

The number of subjects required to identify at least one or more adverse events with a frequency of 10 % and detection rate of >90 % from each group was calculated using the following equation:

(1) *n* = log (1− *p*) / log (1 − *r*); *n*: number of subjects, *r*: frequent of adverse event, *p*: detection rate

From Eq. (1), the required sample size per group was calculated to be 20 subjects. Additionally, two extra subjects were added to each group (22 subjects each) with consideration of dropouts to satisfy a randomized, double-blind, placebo-controlled study as described below.

### 2.4 Enrollment, randomization, and blinding

Among the 63 subjects who submitted signed informed consent, 44 were selected and included in this study. After confirming the indistinguishability between the test foods (C-3102 tablet and placebo), a code was given by the person in charge of shipping from the contract research organization to an allocation controller, who was not directly involved in this study. The allocation controller randomly assigned the subjects to either the C-3102 (*n* = 22) or P group (*n* = 22), whose compositions were nearly equivalent in terms of gender and age. The allocation was performed using StatLight #11 Version 2.10 (Yukms Co., Ltd., Kanagawa, Japan), a computerized random-number generator. The sponsors, principal investigator, sub-investigators, entire staff of the contract research organization (such as the study director, an operation director, monitoring staff, statistical analysis director and staff, and the person in charge of shipping), medical institution staff, institutional review board members, contract laboratory, and other personnel involved in this study were completely blinded to the allocation procedure.

### 2.5 Intervention

All subjects were asked to consume either 18 C-3102 tablets or placebo tablets without chewing every day with water for 4 weeks. The test foods comprised uncoated tablets (8 mm Φ), in which the basic composition was fermented soybean powder (containing 4.8 × 10^10^ cfu of C-3102 per day) and additives, and only placebo food contained additives.

### 2.6 Outcomes

Table 1 shows the study’s schedule. The safety was evaluated before, 2, and 4 weeks after initiating the test food intake.

**Table 1:** Enrollment, intervention, and assessment schedule.

	Study period					
	Enrollment	Screening (baseline)	Allocation	Start intake	Intervention period	
					2 weeks after intake	4 weeks after intake
Enrollment:						
Eligibility screen	**×**					
Informed consent	**×**					
Allocation			**×**			
Interventions:						
C-3102 group						
P group						
Assessments:						
Physical examination		**×**			**×**	**×**
Hematological and blood biochemical test		**×**			**×**	**×**
Urinalysis		**×**			**×**	**×**
Daily record						
Medical questionnaire		**×**			**×**	**×**
Bone density examination		**×**			**×**	**×**

### 2.7 Primary outcomes

The subjects’ height, weight, body mass index, body fat percentage, systolic and diastolic blood pressures, pulse rate, and body temperature were measured during physical examination. Height was only measured at baseline to calculate the body mass index.

Hematological tests were conducted to assess the leukocyte count, erythrocyte count, hemoglobin level, hematocrit value, platelet count, mean corpuscular volume, mean corpuscular hemoglobin, mean corpuscular hemoglobin concentration, and white blood cell differentiation (percentages and total counts of neutrophils, lymphocytes, monocytes, eosinophils, and basophils). Furthermore, biochemical tests evaluated the levels of aspartate transaminase, alanine aminotransferase, γ-glutamyltransferase, alkaline phosphatase, lactate dehydrogenase, leucine aminopeptidase, total bilirubin, direct bilirubin, indirect bilirubin, cholinesterase, zinc turbidity test, total protein, urea nitrogen, creatinine, uric acid, creatine kinase, calcium, serum amylase, total cholesterol, high-density lipoprotein cholesterol, low-density lipoprotein cholesterol, triglycerides, glycoalbumin, serum iron, sodium, potassium, chloride, inorganic phosphorus, glucose, and hemoglobin A1c. Hematological and biochemical tests were performed by LSI Medience Corporation (Tokyo, Japan).

Urine samples were collected to evaluate protein, glucose, urobilinogen, bilirubin, ketone bodies, pH, and occult blood levels, which were also performed by the LSI Medience Corporation.

Subjects were requested to complete a medical questionnaire to determine their health status at each assessment point. Additionally, they were required to report the medication dosage and any changes in their physical condition daily.

### 2.8 Secondary outcome

BMD was calculated using an ultrasound bone densitometer CM-200 (Canon Lifecare Solutions Inc., Tokyo, Japan).

### 2.9 Statistical analysis

Physical examination, urinalysis, blood analysis, and BMD examination data were statically assessed before, 2 weeks after, and 4 weeks after intake (three-time assessment points). The values obtained before intake were considered as baseline values.

The background and demographic data were aggregated based on gender, age, and physical characteristics and compared with those in the P group using the Student’s *t*-test. Physical examination, blood analysis, and BMD examination data were expressed as mean and standard deviation (SD), and baseline values were analyzed using Student’s *t*-test. Physical examination, blood analysis, and BMD examination data at 2 and 4 weeks after intake were analyzed using analysis of covariance (ANCOVA), with covariates used as baseline values. For urinalysis, data were set to a code where 1 and 0 were within and outside the normal range, respectively, and data were expressed as a matrix of the number of subjects (*n*) and the code, followed by the chi-squared test. A subgroup analysis on the red blood cell count, hemoglobin, hematocrit, γ- glutamyltransferase, leucine aminopeptidase, cholinesterase, creatinine, uric acid, creatine kinase, serum iron, and high-density lipoprotein cholesterol was performed due to difference in reference ranges between genders. The subgroup analysis was performed using ANCOVA.

All statistical analyses were two-sided, with a significance level of 5 % with no adjustment for multiple comparisons. Data analysis was performed using Windows SPSS version 23.0 (IBM Japan, Ltd., Tokyo, Japan).

## 3 Results

### 3.1 Setting analysis

**Fig. 1:**
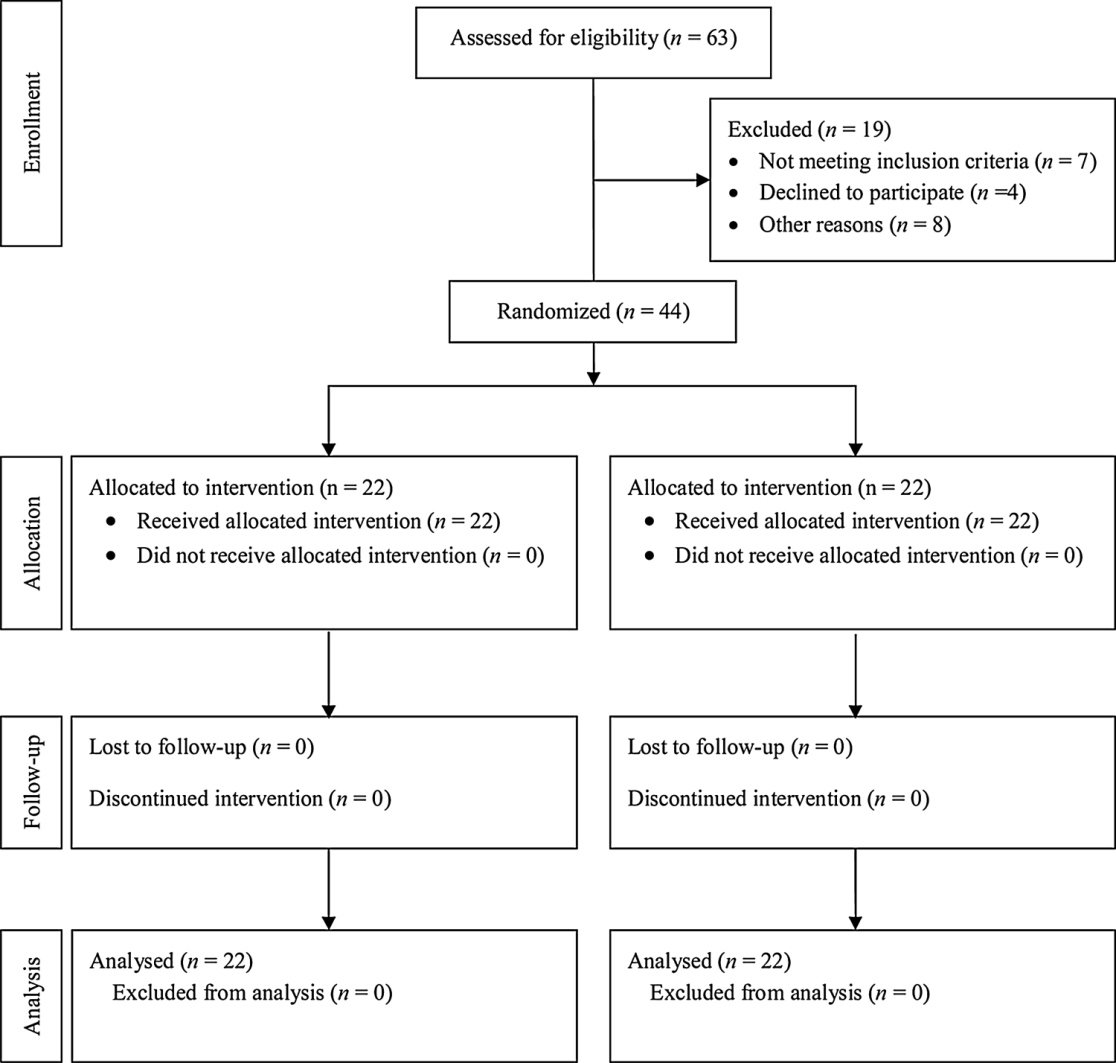
The flowchart of participants in this study.

All subjects completed this study (Fig.1) without violating the protocol and their rates of consumption were >90 %. Therefore, 22 subjects (9 men and 13 women) in the C-3102 group and 22 (9 men and 13 women) in the P group were included in the analysis on an intention-to-treat dataset basis.

The subjects’ demographic characteristics were not statistically significant different between the groups (Table 2a, Table 2b).

**Table 2a:** Subjects’ background information.

Item (unit)	C-3102 group (*n* = 22)		P group (*n* = 22)	
	Mean	SD	Mean	SD
Age (years)	46.1	13.8	46.1	13.5
Height (cm)	164.0	8.8	162.1	8.5
Body weight (kg)	60.2	10.6	59.3	9.5
Body mass index (kg/m^2^)	22.3	2.9	22.6	3.6
Body fat percentage (%)	23.5	6.3	23.8	7.0
Systolic blood pressure (mmHg)	121.4	18.8	113.6	16.1
Diastolic blood pressure (mmHg)	75.0	12.8	71.7	12.6
Pulse rate (bpm)	69.1	10.6	76.7	10.5
Body temperature (°C)	36.2	0.4	36.3	0.3

The data were calculated using Student’s *t-*test.

SD, Standard deviation.

**Table 2b:** Subjects’ background information.

Age (years)	C-3102 group (*n* = 22)		P group(*n* = 22)	
	Men (*n*)	Women (*n*)	Men (*n*)	Women (*n*)
20-29	1	1	1	1
30-39	3	3	3	3
40-49	3	2	3	2
50-59	1	3	1	3
60-69	1	4	1	4
≥70	0	0	0	0

### 3.2 Physical examination

Systolic blood pressure was significantly higher but within the reference range (Table 3). At 2 weeks after consumption, the body fat percentage was significantly lower in the C-3102 group than in the P group (*P* =  0.006, *P* = 0.002, respectively, Table 3).

**Table 3:** Results of physical examination.

Item (Unit)	Baseline				2 weeks				4 weeks				*P* value		
	C-3102 group (*n* = 22)		P group (*n* = 22)		C-3102 group (*n* = 22)		P group (*n* = 22)		C-3102 group (*n* = 22)		P group (*n* = 22)				
	Mean	SD	Mean	SD	Mean	SD	Mean	SD	Mean	SD	Mean	SD	Baseline^a^	2 weeks^b^	4 weeks^b^
Height (cm)	164.0	8.8	162.1	8.5	–		–		–		–		0.486	―	―
Body weight (kg)	60.2	10.6	59.3	9.5	59.7	10.9	59.1	9.4	59.8	11.0	59.3	9.3	0.791	0.509	0.377
Body mass index (kg/m^2^)	22.3	2.9	22.6	3.6	22.1	3.0	22.5	3.6	22.1	3.0	22.6	3.6	0.743	0.515	0.335
Body fat percentage (%)	23.5	6.3	23.8	7.0	23.2	6.7	24.5	6.7	23.2	7.0	24.1	6.6	0.896	0.002**	0.114
Systolic blood pressure (mmHg)	121.4	18.8	113.6	16.1	117.1	14.8	116.4	18.0	118.5	15.9	113.5	17.4	0.149	0.006**	0.360
Diastolic blood pressure (mmHg)	75.0	12.8	71.7	12.6	74.0	10.3	73.5	11.9	73.3	10.0	73.5	13.7	0.394	0.300	0.150
Pulse rate (bpm)	69.1	10.6	76.7	10.5	72.5	8.6	77.7	11.2	71.3	9.0	76.3	10.3	0.022*	0.925	0.916
Body temperature (°C)	36.2	0.4	36.3	0.3	36.1	0.5	36.3	0.3	36.3	0.4	36.2	0.5	0.475	0.289	0.562

The data were calculated using ^a^Student’s *t*-test or ^b^ANCOVA.

SD, Standard deviation.

* *P* < 0.05.

** *P* < 0.01 vs P group.

### 3.3 Blood analysis

#### 3.3.1 All subjects

The mean corpuscular hemoglobin level was significantly higher and cholinesterase, total cholesterol, and triglyceride levels were significantly lower 2 weeks after intake in the C-3102 group than in the P group (*P* =  0.048, *P* =  0.010, *P* =  0.046, and *P* =  0.005, respectively, Table 4a, Table 4b, Table 4c). Moreover, at 4 weeks after intake, direct bilirubin was significantly higher and total cholesterol significantly lower in the C-3102 group than in the P group (*P* =  0.029 and *P* =  0.019, respectively, Table 4b, Table 4c). However, all changes in the C-3102 group were within normal ranges (Table 4a, Table 4b, Table 4c).

**Table 4a:** Results of blood analysis.

Item (Unit)	Reference range	Baseline				2 weeks				4 weeks				*P* value		
		C-3102 group (*n* = 22)		P group (*n* = 22)		C-3102 group (*n* = 22)		P group (*n* = 22)		C-3102 group (*n* = 22)		P group (*n* = 22)				
		Mean	SD	Mean	SD	Mean	SD	Mean	SD	Mean	SD	Mean	SD	Baseline^a^	2 weeks^b^	4 weeks^b^
Leukocyte count (/μL)	3300–9000	5136.4	990.2	5281.8	1375.5	5104.5	888.8	5063.6	1263.2	5218.2	906.9	5209.1	1220.0	0.689	0.699	0.606
Erythrocyte count (×10^4^/μL)	Men: 430–570 Women: 380–500	469.0	44.4	467.1	39.5	463.0	39.6	467.5	36.8	462.1	38.0	460.0	41.1	0.884	0.290	0.930
Hemoglobin level (g/dL)	Men: 13.5–17.5 Women: 11.5–15.0	14.1	1.2	13.9	1.1	14.0	1.1	13.9	1.0	13.9	0.9	13.6	1.2	0.666	0.845	0.522
Hematocrit value (%)	Men: 39.7–52.4 Women: 34.8–45.0	44.1	3.6	43.7	3.1	43.8	3.1	43.7	3.3	44.0	2.6	43.4	3.6	0.644	0.619	0.663
Platelet count (×10^4^/μL)	14.0–34.0	27.7	5.9	26.9	4.4	27.1	6.6	27.9	5.3	27.6	6.0	27.7	4.7	0.654	0.186	0.191
mean corpuscular volume (fL)	85–102	94.3	4.1	93.7	4.7	94.7	3.4	93.5	4.7	95.5	3.7	94.5	4.6	0.635	0.131	0.359
mean corpuscular hemoglobin (pg)	28.0–34.0	30.1	1.1	29.9	1.6	30.2	1.2	29.7	1.5	30.0	1.1	29.6	1.6	0.671	0.048*	0.140
mean corpuscular hemoglobin concentration (%)	30.2–35.1	31.9	1.0	31.9	0.9	31.9	0.9	31.8	0.6	31.5	0.8	31.4	0.8	0.962	0.625	0.521
Percentages of neutrophils (%)	40.0–75.0	55.8	4.9	56.1	7.4	55.4	8.9	55.3	6.8	56.0	7.0	55.8	6.3	0.890	0.931	0.774
Percentages of lymphocytes (%)	18.0–49.0	35.1	5.5	33.7	6.5	35.6	8.0	35.4	6.7	35.7	6.4	35.0	6.4	0.430	0.652	0.713
Percentages of monocytes (%)	2.0–10.0	5.1	1.1	5.8	1.3	5.7	1.7	5.7	1.3	4.8	1.2	5.8	1.6	0.061	0.771	0.167
Percentages of eosinophils (%)	0.0–8.0	3.2	2.2	3.6	3.3	2.7	1.5	2.9	2.0	2.8	1.5	2.6	2.1	0.696	0.962	0.389
Percentages of basophils (%)	0.0–2.0	0.7	0.3	0.9	0.5	0.6	0.3	0.7	0.4	0.7	0.3	0.8	0.5	0.353	0.666	0.883
Neutrophil count (/μL)	–	2865.0	577.3	2981.4	926.5	2872.9	867.4	2838.5	908.1	2929.1	671.3	2931.1	868.0	0.620	0.705	0.627
Lymphocyte count (/μL)	–	1801.3	455.6	1756.3	479.0	1777.9	329.0	1761.3	463.0	1856.9	436.9	1813.0	489.9	0.751	0.921	0.925
Monocyte count (/μL)	–	257.7	59.1	302.5	89.3	289.4	99.0	282.4	67.8	253.9	76.9	293.3	81.6	0.056	0.403	0.519
Eosinophil count (/μL)	–	173.9	136.1	197.3	203.1	133.3	61.2	145.0	112.0	141.6	77.7	129.8	100.1	0.657	0.886	0.305
Basophil count (/μL)	–	38.4	18.2	44.3	24.8	31.2	14.3	36.6	20.2	36.8	17.6	40.0	22.5	0.367	0.605	0.754

The data were calculated using ^a^Student’s *t*-test or ^b^ANCOVA.

SD, Standard deviation.

* *P* <0.05 vs P group.

**Table 4b:** Results of blood analysis.

Item (Unit)	Reference range	Baseline				2 weeks				4 weeks				*P* value		
		C-3102 group (*n* = 22)		P group (*n* = 22)		C-3102 group (*n* = 22)		P group (*n* = 22)		C-3102 group (*n* = 22)		P group (*n* = 22)				
		Mean	SD	Mean	SD	Mean	SD	Mean	SD	Mean	SD	Mean	SD	Baseline^a^	2 weeks^b^	4 weeks^b^
aspartate transaminase (U/L)	10–40	20.2	4.7	20.8	9.1	19.9	4.3	20.4	8.0	19.2	3.8	18.3	5.7	0.789	0.928	0.156
alanine aminotransferase (U/L)	5–45	16.5	5.6	19.0	13.3	17.3	5.6	19.7	15.1	15.6	4.6	16.1	8.7	0.429	0.969	0.493
γ -glutamyltransferase (U/L)	Men: ≤80 Women: ≤30	22.1	15.2	23.0	22.5	21.1	10.9	22.5	17.3	20.6	9.9	23.2	17.2	0.882	0.573	0.162
alkaline phosphatase (U/L)	100–325	213.0	56.0	206.0	56.4	201.5	44.8	204.0	62.9	198.5	47.6	199.5	48.7	0.678	0.309	0.462
lactate dehydrogenase (U/L)	120–240	176.8	29.1	171.6	27.7	176.9	26.6	170.6	22.6	177.5	25.5	172.0	25.1	0.545	0.535	0.700
leucine aminopeptidase (U/L)	Men: 45–81 Women: 37–61	50.4	10.6	49.2	6.7	49.2	8.3	49.9	6.3	49.1	8.6	49.7	7.0	0.649	0.094	0.194
Total bilirubin (mg/dL)	0.2–1.2	1.0	0.4	0.8	0.1	1.0	0.4	0.8	0.2	1.0	0.4	0.8	0.2	0.007**	0.387	0.251
Direct bilirubin (mg/dL)	0.0–0.2	0.1	0.1	0.1	0.0	0.1	0.1	0.1	0.0	0.1	0.0	0.1	0.0	0.227	0.406	0.029*
Indirect bilirubin (mg/dL)	0.2–1.0	0.9	0.4	0.7	0.1	0.9	0.4	0.7	0.2	0.9	0.3	0.7	0.2	0.006**	0.383	0.358
Cholinesterase (U/L)	Men: 234–493	338.8	70.9	334.1	79.0	322.9	69.3	335.5	69.5	324.7	75.2	329.5	67.8	0.837	0.010*	0.212
	Women: 200–452															
zinc turbidity test (U)	2.0–12.0	6.7	2.2	7.2	2.6	6.7	2.2	7.1	2.5	7.4	2.3	7.4	2.6	0.575	0.899	0.178
Total protein (g/dL)	6.7–8.3	7.1	0.4	7.2	0.3	7.0	0.4	7.2	0.2	6.9	0.4	7.1	0.3	0.292	0.194	0.727

The data were calculated using ^a^Student’s *t*-test or ^b^ANCOVA.

SD, Standard deviation.

* *P* <0.05.

** *P* <0.01 vs P group.

**Table 4c:** Results of blood analysis.

Item (Unit)	Reference range	Baseline				2 weeks				4 weeks				*P* value		
		C-3102 group (*n* = 22)		P group (*n* = 22)		C-3102 group (*n* = 22)		P group (*n* = 22)		C-3102 group (*n* = 22)		P group (*n* = 22)				
		Mean	SD	Mean	SD	Mean	SD	Mean	SD	Mean	SD	Mean	SD	Baseline^a^	2 weeks^b^	4 weeks^b^
Urea nitrogen (mg/dL)	8.0–20.0	12.4	2.0	12.6	3.2	12.9	4.7	13.0	3.7	13.7	3.5	12.4	4.0	0.841	0.993	0.143
Creatinine (mg/dL)	Men: 0.61–1.04 Women: 0.47–0.79	0.7	0.2	0.7	0.1	0.7	0.2	0.7	0.1	0.7	0.2	0.6	0.2	0.321	0.741	0.552
Uric acid (mg/dL)	Men: 3.8–7.0 Women: 2.5–7.0	4.9	0.9	4.4	1.4	4.9	1.0	4.5	1.3	4.7	1.0	4.3	1.3	0.169	0.921	0.726
creatine kinase (U/L)	Men: 60–270 Women: 40–150	109.3	66.2	81.1	32.2	98.6	41.9	81.6	42.9	105.3	45.6	73.9	31.9	0.079	0.732	0.071
Sodium (mEq/L)	137–147	141.4	2.1	140.8	1.4	142.4	1.6	141.6	1.4	140.9	1.5	140.2	1.1	0.283	0.225	0.193
Potassium (mEq/L)	3.5–5.0	4.0	0.3	4.1	0.3	3.9	0.3	3.9	0.2	3.9	0.2	3.9	0.3	0.583	0.319	0.814
Chloride (mEq/L)	98–108	102.0	2.1	101.2	2.1	102.2	1.3	101.8	2.0	101.9	1.5	101.7	1.7	0.200	0.912	0.644
Calcium (mg/dL)	8.4–10.4	9.1	0.3	9.2	0.2	9.1	0.2	9.1	0.2	8.9	0.3	9.0	0.3	0.601	0.960	0.965
Inorganic phosphorus (mg/dL)	2.5–4.5	3.4	0.3	3.4	0.5	3.8	0.6	3.6	0.5	3.7	0.4	3.6	0.5	0.509	0.325	0.617
Serum iron (μg/dL)	Men: 50–200 Women: 40–180	112.7	33.6	105.2	39.9	100.8	39.6	112.0	27.3	97.7	20.6	103.8	34.7	0.506	0.255	0.302
Serum amylase (U/L)	40–122	67.6	16.3	70.6	21.3	65.3	17.7	71.1	21.9	68.0	16.1	73.1	24.4	0.603	0.250	0.463
Total cholesterol (mg/dL)	120–219	213.4	42.1	213.4	33.8	204.8	42.5	216.7	27.1	203.6	38.5	215.9	30.1	0.997	0.046*	0.019*
high-density lipoprotein cholesterol (mg/dL)	Men: 40–85 Women: 40–95	69.1	21.4	65.9	11.1	66.8	17.1	65.6	10.0	67.3	20.8	66.7	11.9	0.540	0.483	0.323
low-density lipoprotein cholesterol (mg/dL)	65–139	128.3	32.4	131.0	26.8	124.2	34.3	131.6	24.1	119.9	28.1	129.6	23.6	0.770	0.322	0.073
Triglycerides (mg/dL)	30–149	79.7	46.8	89.5	64.4	81.0	56.1	123.8	84.6	90.6	60.6	122.7	117.4	0.568	0.005**	0.216
Glucose (mg/dL)	70–109	84.4	6.8	83.5	8.6	81.9	6.4	82.5	6.3	82.3	6.1	81.8	6.5	0.715	0.498	0.985
Hemoglobin A1c (%)	4.6–6.2	5.3	0.3	5.4	0.3	5.4	0.2	5.4	0.3	5.4	0.2	5.5	0.3	0.392	0.599	0.221
Glycoalbumin (%)	12.3–16.5	13.5	0.8	13.3	1.0	13.7	0.9	13.6	1.2	14.1	0.8	13.9	1.2	0.573	0.736	0.583

The data were calculated using ^a^Student’s *t*-test or ^b^ANCOVA.

SD, Standard deviation.

* *P* <0.05.

** *P* <0.01 vs P group.

#### 3.3.2 Male subjects

All items of the blood analysis in male subjects did not significantly change between the groups (Table 5).

**Table 5:** Results of blood analysis (men).

Item (Unit)	Reference range	Baseline				2 weeks				4 weeks				*P* value		
		C-3102 group (*n* = 9)		P group (*n* = 9)		C-3102 group (*n* = 9)		P group (*n* = 9)		C-3102 group (*n* = 9)		P group (*n* = 9)				
		Mean	SD	Mean	SD	Mean	SD	Mean	SD	Mean	SD	Mean	SD	Baseline^a^	2 weeks^b^	4 weeks^b^
Erythrocyte count (×10^4^/μL)	430–570	500.6	34.5	493.7	28.5	492.0	29.6	494.1	35.6	460.1	48.8	463.2	35.0	0.650	0.385	0.840
Hemoglobin (g/dL)	13.5–17.5	15.1	0.7	15.0	0.7	14.8	0.7	14.9	0.8	13.5	1.4	13.9	1.1	0.950	0.910	0.433
Hematocrit value (%)	39.7–52.4	46.3	2.3	46.5	2.4	45.7	1.7	46.6	2.6	43.1	4.0	43.9	3.7	0.890	0.343	0.677
γ- glutamyltransferase (U/L)	≤80	26.6	21.1	32.8	32.0	24.0	12.9	31.7	24.0	17.8	5.9	30.1	24.7	0.633	0.073	0.169
leucine aminopeptidase (U/L)	45–81	54.3	14.4	52.0	7.6	52.7	9.7	52.4	7.0	49.1	7.5	51.4	7.0	0.673	0.347	0.434
Cholinesterase (U/L)	234–493	348.3	74.2	381.1	98.9	331.7	65.6	379.3	80.9	300.0	45.1	367.0	81.2	0.438	0.054	0.075
Creatinine (mg/dL)	0.61–1.04	0.9	0.1	0.8	0.1	0.9	0.1	0.8	0.1	0.6	0.2	0.7	0.2	0.135	0.765	0.550
Uric acid (mg/dL)	3.8–7.0	5.3	0.8	5.2	1.5	5.4	0.6	5.3	1.4	4.1	1.4	4.5	1.0	0.863	0.902	0.558
creatine kinase (U/L)	60–270	147.0	83.0	106.3	30.1	120.4	48.2	112.6	47.8	72.8	32.0	80.6	34.7	0.186	0.262	0.831
Serum iron (μg/dL)	50–200	122.1	33.9	94.1	20.1	102.8	32.3	114.7	22.9	103.6	39.8	107.9	34.0	0.049*	0.107	0.924
high-density lipoprotein cholesterol (mg/dL)	40–85	54.0	9.8	65.9	12.2	55.2	9.0	62.4	9.8	65.6	11.4	66.2	13.3	0.036*	0.442	0.785

The data were calculated using ^a^Student’s *t*-test or ^b^ANCOVA.

SD, Standard deviation.

*P <0.05 vs P group..

#### 3.3.3 Female subjects

The cholinesterase levels were significantly higher in female subjects in the C-3102 group than in the P group at 2 weeks after intake (*P* =  0.036, Table 6).

**Table 6:** Results of blood analysis (women).

Item (Unit)	Reference range	Baseline				2 weeks				4 weeks				*P* value		
		C-3102 group (*n* = 13)		P group (*n* = 13)		C-3102 group (*n* = 13)		P group (*n* = 13)		C-3102 group (*n* = 13)		P group (*n* = 13)				
		Mean	SD	Mean	SD	Mean	SD	Mean	SD	Mean	SD	Mean	SD	Baseline^a^	2 weeks^b^	4 weeks^b^
Erythrocyte count (×10^4^/μL)	380–500	447.1	37.3	448.7	36.0	443.0	33.0	449.0	25.0	456.0	43.4	465.3	33.7	0.912	0.474	0.553
Hemoglobin (g/dL)	11.5–15.0	13.4	1.0	13.2	0.6	13.4	1.0	13.2	0.5	13.8	1.0	13.8	1.0	0.460	0.989	0.936
Hematocrit value (%)	34.8–45.0	42.6	3.7	41.7	1.7	42.4	3.1	41.6	1.9	43.6	3.0	44.0	2.3	0.424	0.811	0.726
γ- glutamyltransferase (U/L)	≤30	19.1	9.1	16.2	9.2	19.2	9.3	16.2	5.9	20.5	12.4	20.5	6.6	0.434	0.580	0.909
leucine aminopeptidase (U/L)	37–61	47.7	6.3	47.2	5.4	46.8	6.6	48.1	5.3	49.9	10.6	47.7	5.0	0.843	0.186	0.450
Cholinesterase (U/L)	200–452	332.2	70.8	301.6	40.0	316.8	73.8	305.2	40.8	327.2	89.4	318.1	48.9	0.187	0.036*	0.466
Creatinine (mg/dL)	0.47–0.79	0.6	0.1	0.6	0.1	0.6	0.1	0.6	0.1	0.7	0.2	0.7	0.2	0.457	0.643	0.638
Uric acid (mg/dL)	2.5–7.0	4.6	0.8	3.8	1.0	4.5	1.0	3.9	0.9	4.7	1.2	4.5	1.1	0.042*	0.872	0.598
creatine kinase (U/L)	40–150	83.2	35.8	63.6	20.1	83.5	30.3	60.2	21.9	86.0	33.6	111.2	53.9	0.098	0.191	0.215
Serum iron (μg/dL)	40–180	106.2	33.2	112.9	48.6	99.4	45.1	110.2	30.7	96.1	19.0	98.5	25.0	0.682	0.497	0.888
high-density lipoprotein cholesterol (mg/dL)	40–95	79.5	21.2	65.9	10.8	74.8	17.1	67.8	10.0	65.1	18.8	70.5	20.7	0.050	0.266	0.871

The data were calculated using ^a^Student’s *t*-test or ^b^ANCOVA.

SD, Standard deviation.

* *P <0.05* vs P group.

### 3.4 Urinalysis

There were no significant differences between the groups (Table 7).

**Table 7:** Urinalysis results.

Item		C-3102 group (*n* = 22)		P group (*n* = 22)		*P* value
		Reference range		Reference range		
		Within (*n*)	Outside (*n*)	Within (*n*)	Outside (*n*)	
Protein	Baseline	20	2	20	2	1.00
	2 weeks	22	0	18	4	0.11
	4 weeks	22	0	21	1	1.00
Glucose	Baseline	22	0	22	0	N.A.
	2 weeks	22	0	22	0	N.A.
	4 weeks	22	0	22	0	N.A.
Urobilinogen	Baseline	22	0	22	0	N.A.
	2 weeks	22	0	22	0	N.A.
	4 weeks	22	0	22	0	N.A.
Bilirubin	Baseline	22	0	22	0	N.A.
	2 weeks	22	0	22	0	N.A.
	4 weeks	22	0	22	0	N.A.
pH	Baseline	22	0	21	1	1.00
	2 weeks	22	0	22	0	N.A.
	4 weeks	22	0	21	1	1.00
Occult blood	Baseline	19	3	22	0	0.23
	2 weeks	19	3	19	3	1.00
	4 weeks	21	1	22	0	1.00
Ketone bodies	Baseline	21	1	21	1	1.00
	2 weeks	21	1	22	0	1.00
	4 weeks	22	0	22	0	N.A.

Data are presented as number of participants and was calculated using the chi-squared test.

N.A.: not applicable.

### 3.5 Medical questionnaire and daily report

No physical condition change related to test foods was recorded in the medical questionnaire and daily report written by the subjects (data not shown).

### 3.6 BMD

No significant differences were observed in BMD between groups (Table 8).

**Table 8:** Bone density results.

Item (Unit)	Baseline				2 weeks				4 weeks				*P* value		
	C-3102 group (*n* = 22)		P group (*n* = 22)		C-3102 group (*n* = 22)		P group (*n* = 22)		C-3102 group (*n* = 22)		P group (*n* = 22)				
	Mean	SD	Mean	SD	Mean	SD	Mean	SD	Mean	SD	Mean	SD	Baseline^a^	2 weeks^b^	4 weeks ^b^
Bone density (m/sec)	1513.3	30.4	1500.2	24.1	1513.7	36.5	1503.2	26.4	1512.2	35.1	1502.3	25.4	0.119	0.449	0.427

The data were calculated using ^a^Student’s *t*-test or ^b^ANCOVA.

SD, Standard deviation.

## 4 Discussion

This study investigated the safety of C-3102 tablet intake for 4 weeks in healthy Japanese adult subjects. Both groups took the appropriate 18 tablets of either C-3102 (4.8 × 10^10^ cfu) or placebo per day. The safety was evaluated through physical examinations, urinalysis, blood analysis, and BMD measurement.

Regarding the physical examination, differences in pulse rate were observed at baseline. Although systolic blood pressure and body fat percentage were significantly different 2 weeks after intake, the fluctuation in systolic blood pressure was within the normal range prescribed in the Japanese Society of Hypertension Guidelines for the Management of Hypertension [9], and the fluctuation in body fat percentage was minor with no medically problematic changes in physical conditions during the intervention period.

The blood analysis results revealed that total bilirubin and indirect bilirubin were significantly different between the groups at baseline. Further, significant differences in mean corpuscular hemoglobin, cholinesterase, total cholesterol, and triglyceride were observed 2 weeks after intake, and direct bilirubin and total cholesterol were significantly different 4 weeks after intake between the groups. Regarding the subgroup analyses for gender-dependent measurements, the male group presented significant differences in serum iron and high-density lipoprotein cholesterol at baseline between the groups. In contrast, the blood analysis results in women revealed a significant difference in uric acid at baseline and in cholinesterase 2 weeks after intake between the groups. However, despite significant intergroup differences observed in the blood analysis, all the average values measured were within the reference range in both the C-3102 and P groups; thus, we concluded that no harmful effects were caused by the test food intakes.

Urinalysis revealed no significant differences between the groups. Individual data review revealed that protein, pH, occult blood, and ketone body levels were higher/lower than the reference range in some subjects; however, all were identified to have no complication in the following comprehensive consideration: one subject in the P group had pH values outside the reference range at baseline and 2 weeks after intake. However, no complication was found in this subject, including any medical problem in physical condition. Although one subject in each group had ketone body values outside the reference range at baseline, no problem in other results were identified and they continued to participate in this study. Protein data showed positive or false-positive results in two subjects in the C-3102 group and four subjects in the P group between the baseline and intervention period. A previous study reported that the urinary protein-positive rate represents the average in all ages and that the rate in men is 1.8-fold higher than that in women [10]. Furthermore, the positive rate in urinary protein can be caused by mental stress, bathing in hot water, and orthostatic albuminuria [11]. Based on medical observations and examination results, subjects with positive or false-positive results were permitted to continue participating in this study. Five and three subjects in the C-3102 and P groups showed positive or false-positive results for occult blood, and all were women. Among the eight subjects with positive or false-positive results for occult blood, two were confirmed to be possibly affected by menstruation, and the remaining six exhibited positive or false-positive results despite menopausal or non-menstrual period. However, women typically have a higher positive rate of urine occult blood than women at any age [12], and urine red blood cell count in women is twice higher than that in men, even without abnormal findings [13]. Additionally, a previous study reported that the occult blood positive rate increases with age [14]. Based on these medical examination results, subjects with positive or false-positive results were permitted to continue to participate in this study, and none of these positive or false-positive results were considered to be caused by test foods.

Regarding BMD, no significant differences were observed during the intervention period. Furthermore, ingestion of the test foods did not decrease BMD.

In summary, although significant differences between the C-3102 and P groups were observed in several measurements, these values remained within the reference ranges and did not indicate any complication in the subjects’ conditions. Furthermore, no adverse events were found following physician consultations and review of subjects’ self-records during the intervention period. Therefore, consumption of an excessive amount of C-3102 tablets determined to be safe.

## 5 Conclusion

This study assessed the safety of administering 4.8 × 10^10^ cfu of C-3102 tablets daily for 4 weeks on healthy Japanese adult subjects. The results clearly demonstrated that the intake of C-3102 tablets was safe.

## Funder

Asahi Calpis Wellness Co., Ltd.

## Transparency document

Transparency document

This study was sponsored by the following companies: Asahi Calpis Wellness Co., Ltd funded the study implementation and manuscript writing. M. H., H. K., R. T., I. N., and M. Y. are members of Asahi Calpis Wellness Co., Ltd. that entrusted ORTHOMEDICO, Inc. T. H. and S. I., who are members of ORTHOMEDICO Inc., were involved in the planning of the study, the study implementation, and manuscript writing. The study was conducted by both Asahi Calpis Wellness Co., Ltd and ORTHOMEDICO Inc. Furthermore, T. T. (MD) is the principal investigator who monitors all subjects’ conditions.

## Declaration of Competing Interest

This study was sponsored by the following companies: Asahi Calpis Wellness Co., Ltd funded the study implementation and manuscript writing. M. H., H. K., R. T., I. N., and M. Y. are members of Asahi Calpis Wellness Co., Ltd. that entrusted ORTHOMEDICO, Inc. T. H. and S. I., who are members of ORTHOMEDICO Inc., were involved in the planning of the study, the study implementation, and manuscript writing. The study was conducted by both Asahi Calpis Wellness Co., Ltd and ORTHOMEDICO Inc. Furthermore, T. T. (MD) is the principal investigator who monitors all subjects’ conditions.

## Footnotes

1 # Abbreviations; C-3102, *Bacillus subtilis* C-3102; BMD, bone mineral density
